# An experimental demonstration that early-life competitive disadvantage accelerates telomere loss

**DOI:** 10.1098/rspb.2014.1610

**Published:** 2015-01-07

**Authors:** Daniel Nettle, Pat Monaghan, Robert Gillespie, Ben Brilot, Thomas Bedford, Melissa Bateson

**Affiliations:** 1 Centre for Behaviour and Evolution and Institute of Neuroscience, Newcastle University, Newcastle, UK; 2 Institute of Biodiversity, Animal Health and Comparative Medicine, University of Glasgow, Glasgow, UK; 3 School of Biological Sciences, Plymouth University, Plymouth, UK

**Keywords:** telomeres, early-life adversity, early-life stress, oxidative stress, starlings, *Sturnus vulgaris*

## Abstract

Adverse experiences in early life can exert powerful delayed effects on adult survival and health. Telomere attrition is a potentially important mechanism in such effects. One source of early-life adversity is the stress caused by competitive disadvantage. Although previous avian experiments suggest that competitive disadvantage may accelerate telomere attrition, they do not clearly isolate the effects of competitive disadvantage from other sources of variation. Here, we present data from an experiment in European starlings (*Sturnus vulgaris*) that used cross-fostering to expose siblings to divergent early experience. Birds were assigned either to competitive advantage (being larger than their brood competitors) or competitive disadvantage (being smaller than their brood competitors) between days 3 and 12 post-hatching. Disadvantage did not affect weight gain, but it increased telomere attrition, leading to shorter telomere length in disadvantaged birds by day 12. There were no effects of disadvantage on oxidative damage as measured by plasma lipid peroxidation. We thus found strong evidence that early-life competitive disadvantage can accelerate telomere loss. This could lead to faster age-related deterioration and poorer health in later life.

## 1. Introduction

Environmental conditions experienced early in life can exert powerful delayed effects on adult survival and health, both in humans and in other animals [1,2]. The evidence suggests such effects can be caused by early nutritional restriction, but also by the experience of social stress even if nutrition is adequate [3–5]. An important pathway by which adversity in early life might become lastingly embedded in the individual's phenotype is via effects on telomeres [6]. Telomeres are repetitive non-coding DNA sequences at the ends of eukaryotic chromosomes that play an important role in protecting genomic integrity [7]. Telomeres shorten with age, and short telomere length or rapid telomere loss prospectively predicts individual survival across a range of taxa (e.g. [8–10]).

A number of studies in humans have found relatively short telomeres in individuals who were exposed to early-life environmental adversity [11–13], but these studies are necessarily correlational. In altricial birds, it is possible to use cross-fostering to experimentally manipulate the early environment an individual experiences. Assigning pairs of siblings to different host nests allows genetic background to be held constant while social conditions are varied. Thus, cross-fostering provides a powerful paradigm for identifying the effects of early experience on adult phenotype [14–16]. So far, researchers have investigated effects of early adversity on telomeres in altricial birds by manipulating brood size [17–19]. Two studies have found accelerated telomere attrition in chicks in large broods, but this was more pronounced in [18], or restricted to [19], the smaller chicks within the large broods. This suggests that what is driving the effects may not be brood size *per se*, but rather, disadvantage in within-brood competition. The main aim of this study was therefore to cleanly manipulate competitive disadvantage *without* varying brood size, to examine its impact on telomere dynamics through early life.

One of the key mechanisms thought to be involved in accelerated telomere shortening is oxidative stress exposure. Oxidative stress reflects the net cellular effects of reactive oxygen species and antioxidant mechanisms [20]. Physiological stress increases oxidative damage [21,22], providing one potential pathway from the experience of early-life adversity to increased telomere attrition. Telomeric DNA is rich in guanine and particularly susceptible to oxidative damage. There is both *in vitro* and *in vivo* evidence that oxidative stress accelerates telomere shortening [23–26]. A subsidiary aim of this study was therefore to test whether any effects of competitive disadvantage in the nest on telomere dynamics were correlated with increased oxidative stress exposure.

With these aims in mind, we designed a brood manipulation in wild starling chicks in which we varied position in the within-brood hierarchy, but not brood size. The manipulation involved taking quartettes of focal siblings on post-hatching day 3 (D3, where hatching is D1), and cross-fostering two of them to a brood where all the other chicks were slightly smaller than the focal (the advantage or ADV condition), and the other two to a brood with the same number of chicks, but where all the other chicks were slightly larger than the focal (the disadvantage or DIS condition). This manipulation has a basis in the natural hatching sequence of starlings, where there is often one egg that hatches a day later than the others [27]. The smaller chicks that hatch from such eggs have higher rates of mortality than the rest of the brood. Smaller chicks within a starling brood tend to be jostled to less favourable positions in the chick mass [28]. They beg more intensely, and yet still tend to receive less parental investment than their competitors do [28]. We then compared growth, telomere dynamics and oxidative stress of the siblings in the two conditions. As the two sexes have often been found to be differentially affected by early competition or food restriction in passerine birds [29–31], we examined the effects of sex and interactions between sex and experimental treatment on all of our outcome measures.

## 2. Material and methods

### (a) Subjects and fate

Subjects were wild European starling chicks, *Sturnus vulgaris*, hatched in nest-boxes on four farms in Northumberland, England. A total of 132 chicks were cross-fostered and weighed during the course of the study. Of these, 48 chicks from 12 broods were chosen as focal birds and were subjected to blood sampling and either the DIS or ADV treatment (see §2*b* for details). The remaining 84 birds formed the nest competitors of the focal birds but were not blood sampled and were left in the wild to fledge naturally. Of the initial sample of 48 focal birds, 38 were taken into captivity on post-hatching day 12 (D12, where hatching is D1). The remaining 10 focals had either died before D12 (two DIS and three ADV birds), or were left to fledge naturally because several other members of their natal family had died (four DIS and one ADV birds). The 38 focal birds that were brought into captivity remain living in aviaries at Newcastle University, apart from one that died in October 2013 and another in June 2014.

### (b) Brood-hierarchy cross-fostering manipulation

All chicks hatching in the nest-boxes in the study area between 5 May and 13 May 2013 were weighed using a portable digital balance on D2. We identified focal quartettes of siblings that were all of similar D2 weight. We then composed broods from all the chicks available that day, such that two of each focal quartette would be placed in a brood where they were the largest chicks (the advantaged or ADV condition), while the other two would be placed in a brood where they were the smallest (the disadvantaged or DIS condition). The composed brood size was always the same for the ADV and the DIS half of a focal quartette (in one case, four chicks; in four cases, five chicks; in seven cases, six chicks).

ADV and DIS birds did not differ significantly from one another in D2 weights (means + s.d.: ADV 12.613 + 2.407 g, DIS 12.521 + 2.273 g; *B* = −0.092, s.e.(*B*) = 0.427, likelihood ratio test (LRT) = 0.048, *p* = 0.827). ADV birds were heavier than the mean of their competitors by an average of 4.864 g on D2 (s.d. 1.918 g), while DIS birds were lighter than the mean of their non-focal competitors by an average of 4.793 g on D2 (s.d. 2.196). All focal chicks were weighed on D3, D4, D7 and D12. In addition, all non-focal competitors from each nest containing focal birds were weighed on D4, D7 and D12.

### (c) Captivity and hand-rearing

On D12 after weighing, focal chicks were placed in their quartettes in covered buckets containing nest material and transported by road to the laboratory (maximum 90 min). On arrival, the birds were hand-reared to independence. Each quartette was kept in its covered bucket in a laboratory with low light and a 15 L : 9 D cycle. All birds began to beg within 24 h of arrival, and thereafter were fed to satiety using tweezers as often as they begged. Diet consisted of commercially available poultry-based cat biscuits soaked in water, shredded chicken breast marketed as cat food, and pureed apple. Birds in captivity were weighed on D15, D18, D21 and D24. In addition, at D24, all chicks had both tarsae measured using digital callipers. Tarsus measurements had high repeatability (0.867 *sensu* [32]), and tarsus length reported herein is the mean of the two measurements.

Chicks began to fledge from D20 onwards by flying from their buckets as the lid was lifted. At this point, they were placed in cages 150 × 45 × 45 cm (h × w × d) containing baths and four wooden perches. The birds had imprinted on the experimenters and begged for food in the cages, which was delivered through the cage door. On D24 after weighing and blood sampling, birds were transferred to large free-flight aviaries enriched with sawdust substrate, ropes, baths and suspended boxes that are their permanent homes. Ad libitum food was available from this point, although the birds did not immediately forage for themselves, and continued to come to experimenters to be fed from tweezers for up to 10 days after D24.

### (d) Blood sampling

Owing to the small size of the chicks, we were unable to take a baseline blood sample before the beginning of the experimental manipulation, so we took a first sample on D3 (24 h into the manipulation), and a second at the end of the experimental manipulation (D12). In addition, we took a blood sample at independence (D24) in order to establish whether any treatment differences in telomere length had endured beyond the end of the experimental manipulation. For each blood sample, we extracted 75 µl of blood using a sterile needle and a heparinized capillary tube to collect the blood (D3, medial metatarsal vein; D12 and D24, ulnar vein). Blood samples were taken in a warm car during the field period and a laboratory room after coming into captivity. No bird suffered detectable adverse consequences from blood sampling. Samples were immediately placed on ice, and within 2 h centrifuged to separate cells from plasma. Immediately after this, cells and plasma samples were frozen to −80°C. Blood samples were taken on D3, D12 and D24, and all samples were analysed simultaneously, with samples from the same individual on the same plate.

### (e) Telomere analysis

Genomic DNA was extracted from red blood cells using the MACHEREY-NAGEL Nucleospin Blood Kit (MACHEREY-NAGEL GmbH & Co. KG, Düren, Germany) by resuspending 3–4 µl of red blood cells in 196 μl of PBS and following the manufacturer's protocol for DNA purification from whole blood. The concentration and quality of DNA samples were assessed using a Nanodrop-8000 spectrophotometer; only samples with A260/280 > 1.8 and an A260/230 > 1.9 were assayed. DNA samples were stored at −20°C.

Relative telomere measurements were made using the qPCR method as described by Criscuolo *et al*. [33]. This method is well suited for the examination of within-individual changes in telomere length [34]. It provides a ratio of the abundance of the telomeric sequence to the abundance of the reference single copy gene Gadph (henceforth, the T/S ratio). We made the following modifications to the protocol. DNA samples (10 ng) were assayed using the Absolute blue qPCR SYBR green Low Rox master mix (Thermo scientific) with telomere primers (Tel1b and Tel2b) at a final concentration of 500 nM and Gapdh primers (GapF and GapR) at a final concentration of 70 nM. The telomere thermal profile was 15 min at 95°C, followed by 27 cycles of 15 s at 95°C, 30 s at 58°C, 30 s at 72°C. The Gapdh thermal profile was 15 min at 95°C, followed by 40 cycles of 15 s at 95°C, 30 s at 60°C, 30 s at 72°C. Both assays were followed by melt curve analysis of (58–95°C 1oc/5 s ramp). The reference sample was serially diluted (from 40 to 2.5 ng well^−1^) to produce a standard curve for each plate. This was used to calculate plate efficiencies, all of which fell within the acceptable range (i.e. 100 ± 15%).

Each sample was assayed in triplicate and the mean of the three assays used. Three individuals repeatedly fell outside the Gapdh standard curve and were excluded from the analysis. In total, we obtained D3 T/S ratios from 44 individuals, and D12 and D24 T/S ratios from 35 individuals.

We considered as outcome variables both a measure of telomere length (T/S ratio at D12), and a measure of telomere change (the change in T/S ratio from D3 to D12). For the measure of change, we used Verhulst's *D*, an index that corrects for regression to the mean in imperfectly correlated repeated measurements [35]. (We express *D* so that a negative value indicates attrition and a positive value gain.) The *D* values were highly correlated with the simple difference between T/S at D3 and that at D12 (*r* = 0.961), and all of the results reported below would be the same using the simple difference rather than *D*.

### (f) Oxidative damage

Oxidative stress exposure was measured by assessing damage to lipids via lipid peroxidation in plasma, using malondialdehydes (MDA). MDA is widely employed as a general marker of oxidative stress exposure in the contexts of aging and diseases [36–38]. We quantified MDA in duplicate by high-performance liquid chromatography, following Karatas *et al*. [39], but modifying the volume of sample (5 µl) and reagents [40]. The absorbance of the eluent was monitored at 254 nm and quantified relative to external standards (calibration curves, *r*^2^ = 0.999; repeatability 0.997, *F*_255,254_ = 614.013, *p* < 0.001). Lipid peroxidation was expressed as micrograms of MDA per millilitre of plasma (μg ml^−1^).

### (g) Sex determination

Molecular sexing was carried out by amplification of the chromodomain-helicase-DNA binding (CHD) genes in 10 µl PCR reactions. Final concentrations of reagents were 1× Green GoTaq Flexi buffer (Promega), 2 mM magnesium chloride (Promega), 0.8 mM dNTPs (Promega), 0.8 uM 2550F (5′-GTTACTGATTCGTCTACGAGA-3′) [41], 0.8 µM 2757R (5′-AATTCCCCTTTTATTGATCCATC-3′) (R Griffiths, 2005, unpublished data), 0.375U GoTaq DNA polymerase and approximately 100 ng of DNA. Volumes were brought to 10 µl with H_2_O. The thermal cycle profile for the PCR comprised 94°C for 2 min, followed by 30 cycles of 49°C for 1 min, 72°C for 1 min, 94°C for 45 s, with a final cycle of 49°C for 2 min and 72°C for 5 min. PCR products were separated on a 2% agarose gel, with two bands indicating the presence of a Z and W chromosome (female), and one band indicating the presence of only the Z chromosomes (male). On genetic sexing, it became apparent that there were more males than females in the sample (ADV: 11 males and nine females; DIS: 12 males and six females).

### (h) Data analysis strategy

Data were analysed using R, with package *nlme* [42] for the main statistical models. We fitted linear mixed models using maximum-likelihood estimation. For inference about the significance of parameters, we used the LRT, which compares the change in deviance from dropping the parameter from the model to a *χ*^2^ distribution with 1 d.f. This is the preferred approach to significance testing in linear mixed models [43].

All models included random intercepts for natal family. Including an additional random intercept for host family within natal family would result in a loss of statistical power, as the final sample contained only one bird from several of the host families. However, for the subsample of 28 individuals for which we had complete host family pairs within complete natal family quartettes, we repeated the main analyses reported below with an additional nested random term for host nest. In no case was the model fit significantly improved by the additional random term for host family compared to random term for natal family alone.

For the variables measured at one particular time point, the fixed effects components of the models were Treatment, Sex and the Treatment by Sex interaction. Any additional predictors included are specified in the Results section. No attempt at model reduction was made; reported parameter estimates are always from the full model including all specified terms.

For chick weight gain over time, we fitted a repeated-measures model with a random intercept for bird within natal family, and an autoregressive (AR1) covariance structure. This assumes that there will be a correlation between successive measurements from the same individual. The nonlinearity of the growth curve was handled by inclusion of a Day^2^ term as well as Day. For the fixed effects component of the model for weight gain, we included Day, Day^2^, Treatment, Sex and all the two-way interactions involving Treatment. We also experimented with adding higher order interactions, but none was significant.

## 3. Results

### (a) Effects of experimental manipulation on weight gain and growth

**Figure 1.**
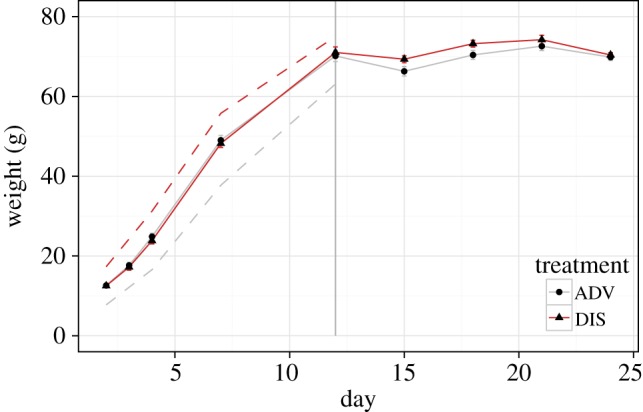
Mean weight (g) over time for birds in the ADV and DIS conditions. Error bars represent 1 s.e. The dashed lines represent the mean weights of the competitors of the focals in each condition. The vertical line identifies the point where the experimental manipulation ended and the birds were taken into captivity. (Online version in colour.)

Figure 1 shows the pattern of weight gain for the ADV birds and their siblings assigned to the DIS treatment. The output of the statistical model for weight gain is summarized in the electronic supplementary material, table S1. There were expected significant effects of Day (*B* = 8.527, s.e.(*B*) = 0.249, LRT = 727.491, *p* < 0.001) and Day^2^ (*B* = −0.230, s.e.(*B*) = 0.010, LRT = 521.452, *p* < 0.001). As figure 1 shows, there were no significant effects of Treatment on weight gain, either as a main effect or in interaction with Day, Day^2^ or Sex. Figure 1 additionally shows the mean weights of the competitors of the ADV and DIS chicks at days 2, 4, 7 and 12. As is clear from the figure, the focal chicks retained their positions relative to their competitors up until D12; larger than their competitors for the ADV chicks, and smaller than their competitors for the DIS chicks.

### (b) Tarsus length at D24

**Figure 2.**
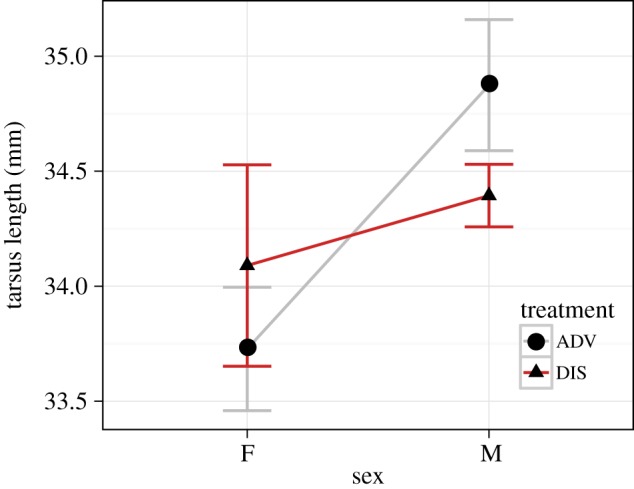
Mean (±s.e.) tarsus length (mm) at independence for birds in the ADV and DIS conditions by sex. (Online version in colour.)

For tarsus length at D24, a measure of skeletal size, there was a significant effect of Sex (*B* = 1.340, s.e.(*B*) = 0.309, LRT = 8.069, *p* = 0.005) and a significant Sex by Treatment interaction (*B* = −1.490, s.e.(*B*) = 0.509, LRT = 7.554, *p* = 0.006; full model output in the electronic supplementary material, Table S2). As figure 2 shows, males (mean + s.d.: 34.562 + 0.799 mm) had longer tarsae than females (mean + s.d.: 33.925 + 0.937 mm) at D24. In addition, ADV birds had longer tarsae (mean + s.d.: 34.407 + 1.029 mm) than their DIS siblings (mean + s.d.: 34.203 + 0.748 mm), but this difference was driven entirely by markedly longer tarsae in the ADV males than the DIS males (ADV males: 34.874 + 0.946 mm; DIS males: 34.394 + 0.471 mm). Thus, competitively disadvantaged females maintained their size, while competitively disadvantaged males did not.

### (c) Oxidative damage

MDA at D3 and D12 were significantly positively correlated (*r*_36_ = 0.431, *p* = 0.007), but MDA was significantly higher at D3 (mean + s.d.: 1668.466 + 584.000) than at D12 (mean + s.d.: 1011.824 + 343.707; paired *t*-test: *t*_27_ = 6.952, *p* < 0.001). The high MDA levels at D3 may have been to do with the extremely rapid growth occurring at this stage. Indeed, there was a significant positive correlation between MDA at D3 and the amount of weight the chick gained from the D3 weighing to the D4 weighing (*r*_45_ = 0.330, *p* = 0.023). MDA at D12 was not correlated with weight at D12 (*r*_36_ = 0.018, *p* = 0.916), or weight gain from D7 to D12 (*r*_36_ = 0–0.071, *p* = 0.670). There were no differences in MDA by Sex, Treatment or the interaction of Sex and Treatment, at either D3 or D12 (electronic supplementary material, Table S3).

### (d) Telomere length and telomere attrition during the manipulation

**Figure 3.**
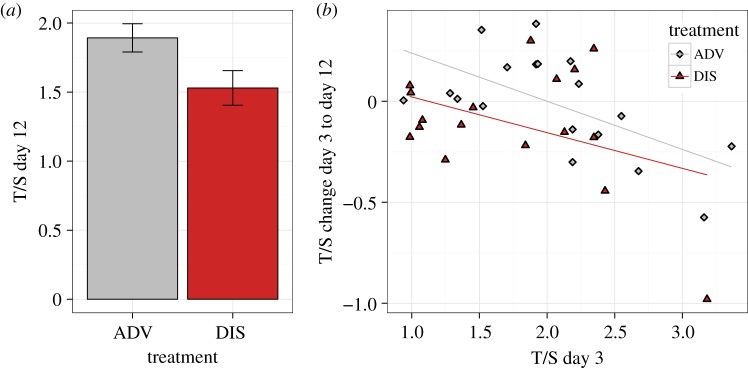
Telomere dynamics over the experimental manipulation. (*a*) Mean T/S at D12 by Treatment. Error bars represent 1 s.e. (*b*) T/S change from D3 to D12 (Verhulst's *D* [35]) by T/S on D3 and Treatment. (Online version in colour.)

Average telomere length as measured by T/S ratio was significantly longer at the beginning of the experimental manipulation than at the end (means + s.d.: D3 1.832 + 0.638; D12 1.716 + 0.503; paired *t*-test: *t*_34_ = 3.341, *p* = 0.002). Electronic supplementary material, Table S4, summarizes the models for T/S ratio at each measurement point. The two treatment groups did not differ significantly in T/S ratio at D3. However, there was a significant effect of Treatment on T/S ratio at D12 (*B* = −0.622, s.e.(*B*) = 0.258, LRT = 5.097, *p* = 0.024), with birds in the DIS condition (mean + s.d.: 1.530 + 0.517) having lower T/S than those in the ADV condition (mean + s.d.: 1.892 + 0.434; figure 3*a*).

The significant difference between treatment groups at D12 where there had been none at D3 suggests that the DIS birds experienced accelerated telomere attrition during the experimental manipulation. To test this directly, we modelled T/S change (corrected for regression to the mean) from D3 to D12 as a function of Treatment, Sex and their interaction. We included T/S at D3 as an additional predictor, as long telomeres are often found to shorten faster than shorter ones, even once regression to the mean has been corrected for [35]. The output from this model is shown in the electronic supplementary material, Table S5. There was a significant effect of T/S at D3, with birds with higher initial T/S showing more attrition (*B* = −0.231, s.e.(*B*) = 0.062, LRT = 12.304, *p* < 0.001). Importantly, there was also a significant effect of treatment, with birds in the DIS condition showing more attrition for their initial T/S than birds in the ADV condition (*B* = −0.232, s.e.(*B*) = 0.134, LRT = 4.795, *p* = 0.029; figure 3*b*).

T/S at D12 and telomere change during the manipulation were not significantly correlated with weight gain during the manipulation (respectively, *r*_33_ = 0.136, *p* = 0.437 and *r*_33_ = −0.037, *p* = 0.832). Adding weight gain to the models predicting T/S D12 or telomere attrition did not improve model fit or alter the effects of Treatment. We also experimented with adding MDA at D3 and at D12 as additional predictors; again, these were not significant and did not alter the conclusions regarding the effect of Treatment.

### (e) Persistence of treatment effects at D24

T/S at D24 was highly correlated with T/S at D12 (*r*_33_ = 0.915, *p* < 0.001). The birds in the DIS condition (mean + s.d.: 1.531 + 0.571) still had a lower mean than those in the ADV condition (mean + s.d.: 1.757 + 0.452). However, although the parameter estimate for Treatment remained negative for T/S at D24 (*B* = −0.562, s.e.(*B*) = 0.275), the effect was no longer significant by the LRT (LRT = 1.756, *p* = 0.185).

## 4. Discussion

Using cross-fostering, we experimentally assigned starling siblings to experience either competitive advantage or competitive disadvantage in the nest between D3 and D12 after hatching. The manipulation had no detectable effect on weight gain; the weights of the siblings in the two different conditions remained extremely similar throughout. This was surprising given previous findings that starling parents preferentially feed the larger chicks in their broods [28]. The breeding season of 2013 was very favourable for starlings, with a warm mild winter meaning that soil invertebrates were abundant. Weight gain for both focal and non-focal birds was rapid and remarkably linear (figure 1). Effects of brood size on growth in starlings are variable from year to year and may not be detectable in particularly favourable years [44], and parental favouritism of larger chicks is context-specific [45]. Thus, the benign conditions may have lifted the constraints that lead to differential allocation of food by chick size. Nonetheless, the weight differences of focal birds relative to their competitors were maintained; the ADV birds were still heavier than their competitors on D12, while the DIS birds were still lighter than their competitors. The linearity of the growth ensured that the initial gap in weights remained approximately constant over time.

Average telomere length, as measured by T/S ratio, shortened significantly over the period of the manipulation. We found evidence for an effect of competitive disadvantage on telomere length. Birds in the DIS condition showed increased attrition, with the consequence that they had significantly lower T/S ratios than the ADV birds by D12. Given the lack of effect of experimental treatment on growth, we can rule out food restriction, or catch-up growth, as explanations for accelerated telomere attrition in the DIS birds. There may have been differences in parental investment between DIS and ADV birds, but they must have been subtle. Previous research suggests that the DIS chicks would have had to beg more to be fed, and would have been pushed to less favourable positions in the chick mass [28]. These factors could lead to elevated levels of physiological stress; elevated stress hormones have been shown experimentally to accelerate early-life telomere loss in birds [46]. However, for logistical reasons, we did not measure stress hormones in this study. In future research, it would be useful to observe parent and chick behaviour within the experimental nests during the manipulation and measure stress hormones directly.

By D24 (after 12 days in captivity), the difference in T/S between the treatment groups was no longer significant, perhaps reflecting the overlain effects of variation in response to hand-rearing experience that was not differentiated by treatment. However, the mean T/S was still lower for DIS birds than their ADV siblings. We also found evidence for greater telomere attrition in birds with higher initial T/S ratios. This phenomenon has often been reported before, but until recently it was not clear whether it simply reflected regression to the mean given imperfect measurement. We used Verhulst's D index of change, which corrects for the expected regression to the mean [35], and we still found significantly greater attrition in individuals with high initial T/S. This concurs with the conclusion that faster attrition in individuals with longer telomeres may be a genuine effect [35,47].

There is discussion in the literature about whether the most relevant measure linking environmental adversity to subsequent survival prospects is telomere length, or the rate of telomere loss [7,18]. In our experiment, the manipulation significantly affected telomere length at D12, but we have shown that it did this through increasing the rate of attrition during the manipulation. We thus found difference at the end of the treatment period both in length and in attrition, the former being brought about via the latter. Where environmental factors accelerate telomere loss, they should also be expected to be associated with final telomere length as long as the contribution of variation in telomere loss to the variation in final telomere length is substantial compared to other influences (e.g. genetic variation in initial telomere length). This is likely to have been the case in our study, due to the sibling design.

We measured oxidative damage levels at the same time points as telomere length, using plasma MDA, a marker of lipid peroxidation. We found oxidative damage to be higher at D3 than D12. This could reflect the stress of being moved, or the rapid growth occurring at this age [48,49]. The relationship between oxidative damage and rapid growth was supported by the fact that those birds that were growing fastest at D3 also had the highest MDA levels. Fast growth is associated with reduced longevity, though the mechanisms underlying this are not well understood [50]. It has been suggested that increased oxidative damage associated with fast growth might play a role [51], but supporting evidence is scarce. Our data do provide some evidence for oxidative damage costs of fast growth. Unlike a previous study in European shags [46], we found no evidence that faster growth was associated with accelerated telomere loss, though the variation in growth rate in our study was, as mentioned above, limited.

We found no evidence that competitive disadvantage increased MDA. We had expected that the increased demands of competition that fall on relatively disadvantaged chicks might lead, via physiological stress, to increased oxidative damage. We also found no evidence that increased MDA levels were associated with more rapid telomere attrition or shorter telomere length. This was contrary to our predictions as there is evidence from both *in vitro* and *in vivo* studies that oxidative stress can accelerate telomere loss [23–26]. However, there are many different kinds of oxidative stress measures, including measures of antioxidant defences in different components of the antioxidant system, and damage to different macromolecules such as proteins, lipids and DNA [20,52]. MDA is a widely used marker of lipid damage and has a number of advantages. It is a measure of actual oxidative damage to cellular lipids; some previous studies have measured antioxidant capacity, but knowing antioxidant capacity alone is not sufficient to infer the level of cellular damage through oxidation [20,53]. MDA has also been shown to correlate well with a wide range of oxidative stress markers of other types [52]. Nonetheless, a complete characterization of oxidative stress would involve multiple measures of damage, whereas we only had a single one in this study. Thus, it would be premature to conclude that oxidative stress exposure is not an important mechanism linking early-life competitive disadvantage to telomere attrition.

We tested for sex-dependent responses to adversity in all our outcome variables. The findings in respect of sex should be viewed with caution due to the small number of females in the sample, particularly in the DIS condition. We found no significant sex differences in MDA or telomere dynamics. Sex differences in telomere dynamics have been reported elsewhere, but the pattern of differences is inconsistent across taxa [54]. The lack of sex differences in telomeres was consistent with our previous study [19]. As for the effects of competitive disadvantage on growth, the weight gain of the two sexes was not differentially affected, but there was a sex-specific response in terms of skeletal size. It was in males but not females that competitive disadvantage lead to smaller tarsus length at independence. Male adult starlings are slightly larger than females, and thus males may have higher energetic demand during the growth period. This may be the reason that the consequences of disadvantage for growth were more pronounced in the males. However, the pattern we observed is at odds with a previous finding on European starlings that parental restriction reduced both skeletal size and weight of female but not male chicks [31]. The reasons for the discrepancy between the studies are not clear, but they highlight the complexity of the interactions that can occur between different types of early adversity and sex.

Overall, our results confirm that subtle variation in social conditions experienced in early life can exert measurable effects on telomeres. Telomere change is at its fastest early in life, and telomere length at the end of growth has been shown to be a significant predictor of subsequent longevity [55]. Thus, the potential for experience-induced changes in telomere dynamics in early life to affect long-term outcomes is strong. Our findings support the utility of telomere dynamics as a sensitive measure linking early experience of stressful situations to adult health and mortality [11–13]. Our results also concur with evidence from previous studies of altricial birds that it is not just brood size that can affect early-life telomere dynamics and that effects of environmental adversity on telomere dynamics are independent of weight gain [18,19]. The relative competitive advantage or disadvantage individuals experienced within their broods, although having no detectable effect on weight gain, had a significant impact on the rate of telomere loss.

## Supplementary Material

Supplementary tables

## Supplementary Material

Data supplement

## Data accessibility

The raw data from the study are downloadable as the electronic supplementary material.

## References

[1] Lindstrom J 1999 Early development and fitness in birds and mammals. Trends Ecol. Evol. 14, 343–348. (10.1016/s0169-5347(99)01639-0) 10441307

[2] Lummaa V Clutton-Brock T 2002 Early development, survival and reproduction in humans. Trends Ecol. Evol. 17, 141–147. (10.1016/S0169-5347(01)02414-4)

[3] Felitti VJ Anda RF Nordenberg D Williamson DF Spitz AM Edwards V Koss MP Marks JS 1998 Relationship of childhood abuse and household dysfunction to many of the leading causes of death in adults—the adverse childhood experiences (ACE) study. Am. J. Prev. Med. 14, 245–258. (10.1016/s0749-3797(98)00017-8) 9635069

[4] Larson K Halfon N 2013 Parental divorce and adult longevity. Int. J. Public Health 58, 89–97. (10.1007/s00038-012-0373-x) 22674375

[5] Lacey RE Kumari M McMunn A 2013 Parental separation in childhood and adult inflammation: the importance of material and psychosocial pathways. Psychoneuroendocrinology 38, 2476–2484. (10.1016/j.psyneuen.2013.05.007) 23838100

[6] Price LH Kao HT Burgers DE Carpenter LL Tyrka AR 2013 Telomeres and early-life stress: an overview. Biol. Psychiatry 73, 15–23. (10.1016/j.biopsych.2012.06.025) 22831981 PMC3495091

[7] Monaghan P 2010 Telomeres and life histories: the long and the short of it. Annu. NY Acad. Sci. 1206, 130–142. (10.1111/j.1749-6632.2010.05705.x) 20860686

[8] Salomons HM Mulder GA van de Zande L Haussmann MF Linskens MHK Verhulst S 2009 Telomere shortening and survival in free-living corvids. Proc. R. Soc. B 276, 3157–3165. (10.1098/rspb.2009.0517) PMC2817122 19520803

[9] Bize P Criscuolo F Metcalfe NB Nasir L Monaghan P 2009 Telomere dynamics rather than age predict life expectancy in the wild. Proc. R. Soc. B 276, 1679–1683. (10.1098/rspb.2008.1817) PMC2660992 19324831

[10] Boonekamp JJ Simons MJP Hemerik L Verhulst S 2013 Telomere length behaves as biomarker of somatic redundancy rather than biological age. Aging Cell 12, 330–332. (10.1111/acel.12050) 23346961

[11] Tyrka AR Price LH Kao HT Porton B Marsella SA Carpenter LL 2010 Childhood maltreatment and telomere shortening: preliminary support for an effect of early stress on cellular aging. Biol. Psychiatry 67, 531–534. (10.1016/j.biopsych.2009.08.014) 19828140 PMC2853238

[12] Shalev I Moffitt TE Sugden K Williams B Houts RM Danese A Mill J Arseneault L Caspi A 2013 Exposure to violence during childhood is associated with telomere erosion from 5 to 10 years of age: a longitudinal study. Mol. Psychiatry 18, 576–581. (10.1038/mp.2012.32) 22525489 PMC3616159

[13] Kananen L Surakka I Pirkola S Suvisaari J Lönnqvist J Peltonen L Ripatti S Hovatta I 2010 Childhood adversities are associated with shorter telomere length at adult age both in individuals with an anxiety disorder and controls. PLoS ONE 5, e10826 (10.1371/journal.pone.0010826) 20520834 PMC2876034

[14] Naguib M Gil D 2005 Transgenerational effects on body size caused by early developmental stress in zebra finches. Biol. Lett. 1, 95–97. (10.1098/rsbl.2004.0277) 17148137 PMC1629067

[15] Riebel K Naguib M Gil D 2009 Experimental manipulation of the rearing environment influences adult female zebra finch song preferences. Anim. Behav. 78, 1397–1404. (10.1016/j.anbehav.2009.09.011)

[16] Verhulst S Holveck M-J Riebel K 2006 Long-term effects of manipulated natal brood size on metabolic rate in zebra finches. Biol. Lett. 2, 478–480. (10.1098/rsbl.2006.0496) 17148435 PMC1686193

[17] Voillemot M Hine K Zahn S Criscuolo F Gustafsson L Doligez B Bize P 2012 Effects of brood size manipulation and common origin on phenotype and telomere length in nestling collared flycatchers. BMC Ecol. 12, 17 (10.1186/1472-6785-12-17) 22901085 PMC3547695

[18] Boonekamp JJ Mulder GA Salomons HM Dijkstra C Verhulst S 2014 Nestling telomere shortening, but not telomere length, reflects developmental stress and predicts survival in wild birds. Proc. R. Soc. B 281, 20133287 (10.1098/rspb.2013.3287) PMC4024283 24789893

[19] Nettle D Monaghan P Boner W Gillespie R Bateson M 2013 Bottom of the heap: having heavier competitors accelerates early-life telomere loss in the European starling, *Sturnus vulgaris*. PLoS ONE 8, e83617 (10.1371/journal.pone.0083617) 24386235 PMC3873947

[20] Monaghan P Metcalfe NB Torres R 2009 Oxidative stress as a mediator of life history trade-offs: mechanisms, measurements and interpretation. Ecol. Lett. 12, 75–92. (10.1111/j.1461-0248.2008.01258.x) 19016828

[21] Epel ES Blackburn EH Lin J Dhabhar FS Adler NE Morrow JD Cawthon RM 2004 Accelerated telomere shortening in response to life stress. Proc. Natl Acad. Sci. USA 101, 17 312–17 315. (10.1073/pnas.0407162101) PMC534658 15574496

[22] Joergensen A Broedbaek K Weimann A Semba RD Ferrucci L Joergensen MB Poulsen HE 2011 Association between urinary excretion of cortisol and markers of oxidatively damaged DNA and RNA in humans. PLoS ONE 6, e20795 (10.1371/journal.pone.0020795) 21687734 PMC3110199

[23] von Zglinicki T 2002 Oxidative stress shortens telomeres. Trends Biochem. Sci. 27, 339–344. (10.1016/s0968-0004(02)02110-2) 12114022

[24] Richter T Zglinicki TV 2007 A continuous correlation between oxidative stress and telomere shortening in fibroblasts. Exp. Gerontol. 42, 1039–1042. (10.1016/j.exger.2007.08.005) 17869047

[25] Cattan V 2008 Chronic oxidative stress induces a tissue-specific reduction in telomere length in CAST/Ei mice. Free Radic. Biol. Med. 44, 1592–1598. (10.1016/j.freeradbiomed.2008.01.007) 18249196

[26] Houben JMJ Moonen HJJ van Schooten FJ Hageman GJ 2008 Telomere length assessment: biomarker of chronic oxidative stress? Free Radic. Biol. Med. 44, 235–246. (10.1016/j.freeradbiomed.2007.10.001) 18021748

[27] Feare C 1984 The starling. Oxford, UK: Oxford University Press.

[28] Cotton PA Wright J Kacelnik A 1999 Chick begging strategies in relation to brood hierarchies and hatching asynchrony. Am. Nat. 153, 412–420. (10.1086/303178) 29586619

[29] Råberg L Stjernman M Nilsson JÅ 2005 Sex and environmental sensitivity in blue tit nestlings. Oecologia 145, 496–503. (10.1007/s00442-005-0133-1) 15965752

[30] De Kogel CH 1997 Long-term effects of brood size manipulation on morphological development and sex-specific mortality of offspring. J. Anim. Ecol. 66, 167–178. (10.2307/6019)

[31] Rowland E Love OP Verspoor JJ Sheldon L Williams TD 2007 Manipulating rearing conditions reveals developmental sensitivity in the smaller sex of a passerine bird, the European starling *Sturnus vulgaris*. J. Avian Biol. 38, 612–618. (10.1111/j.0908-8857.2007.04082.x)

[32] Lessells CM Boag PT 1987 Unrepeatable repeatabilities: a common mistake. Auk 104, 116–121. (10.2307/4087240)

[33] Criscuolo F Bize P Nasir L Metcalfe NB Foote CG Griffiths K Gault EA Monaghan P 2009 Real-time quantitative PCR assay for measurement of avian telomeres. J. Avian Biol. 40, 342–347. (10.1111/j.1600-048X.2008.04623.x)

[34] Nussey DH 2014 Measuring telomere length and telomere dynamics in evolutionary biology and ecology. Methods Ecol. Evol. 5, 299–310. (10.1111/2041-210X.12161) 25834722 PMC4375921

[35] Verhulst S Aviv A Benetos A Berenson GS Kark JD 2013 Do leukocyte telomere length dynamics depend on baseline telomere length? An analysis that corrects for ‘regression to the mean’. Eur. J. Epidemiol. 28, 859–866. (10.1007/s10654-013-9845-4) 23990212

[36] Kasapoglu M Özben T 2001 Alterations of antioxidant enzymes and oxidative stress markers in aging. Exp. Gerontol. 36, 209–220. (10.1016/S0531-5565(00)00198-4) 11226737

[37] Gil L Siems W Mazurek B Gross J Schroeder P Voss P Grune T 2006 Age-associated analysis of oxidative stress parameters in human plasma and erythrocytes. Free Radical Res. 40, 495–505. (10.1080/10715760600592962) 16551576

[38] Díaz-Vélez CR García-Castiñeiras S Mendoza-Ramos E Hernández-López E 1996 Increased malondialdehyde in peripheral blood of patients with congestive heart failure. Am. Heart J. 131, 146–152. (10.1016/S0002-8703(96)90063-0) 8554002

[39] Karatas F Karatepe M Baysar A 2002 Determination of free malondialdehyde in human serum by high-performance liquid chromatography. Anal. Biochem. 311, 76–79. (10.1016/S0003-2697(02)00387-1) 12441155

[40] Noguera JC Alonso-Alvarez C Kim SY Morales J Velando A 2011 Yolk testosterone reduces oxidative damages during postnatal development. Biol. Lett. 7, 93–95. (10.1098/rsbl.2010.0421) 20659922 PMC3030863

[41] Fridolfsson AK Ellegren H 1999 A simple and universal method for molecular sexing of non-ratite birds. J. Avian Biol. 30, 116–121. (10.2307/3677252)

[42] Pinheiro J Bates D DebRoy S Sarkar D 2013 nlme: linear and nonlinear mixed effects models. (3.1-111 ed, R Core Development Team. See http://cran.r-project.org/.

[43] Faraway JJ 2006 Extending the linear model with R: generalized linear, mixed effects and non parametric regression models. Boca Raton, FL: Chapman and Hall/CRC.

[44] Westerterp K 1973 The energy budget of the nestling starling *Sturnus vulgaris*: a field study. Ardea 61, 137–158.

[45] Bize P Piault R Moureau B Heeb P 2006 A UV signal of offspring condition mediates context-dependent parental favouritism. Proc. R. Soc. B 273, 2063–2068. (10.1098/rspb.2006.3546) PMC1635472 16846914

[46] Herborn KA Heidinger BJ Boner W Noguera JC Adam A Daunt F Monaghan P 2014 Stress exposure in early post-natal life reduces telomere length: an experimental demonstration in a long-lived seabird. Proc. R. Soc. B 281, 20133151 (10.1098/rspb.2013.3151) PMC3973262 24648221

[47] Bauch C Becker PH Verhulst S 2014 Within the genome, long telomeres are more informative than short telomeres with respect to fitness components in a long-lived seabird. Mol. Ecol. 23, 300–310. (10.1111/mec.12602) 24274429

[48] Geiger S Le Vaillant M Lebard T Reichert S Stier A Le Maho Y Criscuolo F 2012 Catching-up but telomere loss: half-opening the black box of growth and ageing trade-off in wild king penguin chicks. Mol. Ecol. 21, 1500–1510. (10.1111/j.1365-294X.2011.05331.x) 22117889

[49] Rollo CD Carlson J Sawada M 1996 Accelerated aging of giant transgenic mice is associated with elevated free radical processes. Can. J. Zool. 74, 606–620. (10.1139/z96-070)

[50] Metcalfe NB Monaghan P 2003 Growth versus lifespan: perspectives from evolutionary ecology. Exp. Gerontol. 38, 935–940. (10.1016/S0531-5565(03)00159-1) 12954479

[51] Metcalfe NB Alonso-Alvarez C 2010 Oxidative stress as a life-history constraint: the role of reactive oxygen species in shaping phenotypes from conception to death. Funct. Ecol. 24, 984–996. (10.1111/j.1365-2435.2010.01750.x)

[52] Dotan Y Lichtenberg D Pinchuk I 2004 Lipid peroxidation cannot be used as a universal criterion of oxidative stress. Prog. Lipid Res. 43, 200–227. (10.1016/j.plipres.2003.10.001) 15003395

[53] Costantini D Verhulst S 2009 Does high antioxidant capacity indicate low oxidative stress? Funct. Ecol. 23, 506–509. (10.1111/j.1365-2435.2009.01546.x)

[54] Barrett ELB Richardson DS 2011 Sex differences in telomeres and lifespan. Aging Cell 10, 913–921. (10.1111/j.1474-9726.2011.00741.x) 21902801

[55] Heidinger BJ Blount JD Boner W Griffiths K Metcalfe NB Monaghan P 2012 Telomere length in early life predicts lifespan. Proc. Natl Acad. Sci. USA 109, 1743–1748. (10.1073/pnas.1113306109) 22232671 PMC3277142

