# Supplementary material for: An experimental demonstration that early-life competitive disadvantage accelerates telomere loss
Source: Proc Biol Sci. 2015 Jan 7;282(1798):20141610. doi: 10.1098/rspb.2014.1610 (PMC4262165; doi:10.1098/rspb.2014.1610)
Supplement: Supplementary tables [file rspb20141610supp1.pdf]

Supplementary Tables for Nettle et al., 'An experimental demonstration that early-life competitive disadvantage accelerates telomere loss'

Supplementary Table 1. Model output for weight gain up to D24.

Fixed effects	*Parameter estimate*	*s.e.*	*LRT*
Intercept	-5.103	1.389	
Day	8.527	0.249	727.491*
Day^2	-0.230	0.010	521.452*
Sex (M)	1.517	1.089	1.122
Treatment (DIS)	-0.706	1.990	1.022
Treatment * Day	0.484	0.356	1.875
Treatment * Day^2	-0.016	0.014	1.300
Treatment * Sex	-1.581		0.852
Within-individual autocorrelation		0.388	
Variances			
Natal family		0.690	
Bird within family		0.001	
Residual		26.303	

* $p < 0.05$

Supplementary Table 2. Model output for tarsus length at D24.

Fixed effects	*Parameter estimate*	*s.e.*	*LRT*
Intercept	33.621	0.281	
Sex (M)	1.340	0.309	8.069*
Treatment (DIS)	0.753	0.369	0.508
Treatment*Sex	-1.490	0.509	7.554*
Variances			
Natal family		0.289	
Residual		0.320	

* $p < 0.05$

Supplementary Table 3. Model output for oxidative damage (MDA) at D3 and D12.

Day 3	*Fixed effects*	*Parameter estimate*	*s.e.*	*LRT*
	Intercept	1789.195	182.766	
	Sex (M)	-106.299	150.483	0.024
	Treatment (DIS)	-291.469	177.901	2.347
	Treatment*Sex	259.272	254.320	1.119
	Variances			
	Natal family		240201.5	
	Residual		83852.5	
Day 12	*Fixed effects*	*Parameter estimate*	*s.e.*	*LRT*
	Intercept	973.874	117.520	
	Sex (M)	198.739	146.728	1.829
	Treatment (DIS)	-84.265	173.855	3.101
	Treatment*Sex	-140.321	234.002	0.396
	Variances			
	Natal family		25358.3	
	Residual		79906.7	

* $p < 0.05$

Supplementary Table 4. Model output for telomere length (T/S) at D3 and D12.

Day 3	*Fixed effects*	*Parameter estimate*	*s.e.*	*LRT*
	Intercept	2.100	0.209	
	Sex (M)	-0.242	0.271	0.148
	Treatment (DIS)	-0.628	0.295	1.803
	Treatment*Sex	0.629	0.384	2.855
	Variances			
	Natal family		≈0	
	Residual		0.356	
Day 12	*Fixed effects*	*Parameter estimate*	*s.e.*	*LRT*
	Intercept	1.976	0.170	
	Sex (M)	-0.147	0.227	0.101
	Treatment (DIS)	-0.622	0.258	5.097*
	Treatment*Sex	0.425	0.336	1.700
	Variances			
	Natal family		0.007	
	Residual		0.196	

* $p < 0.05$

Supplementary Table 5. Model output for telomere attrition (D) between D3 and D12

Fixed effects	*Parameter estimate*	*s.e.*	*LRT*
Intercept	0.516	0.160	
T/S D3	-0.231	0.062	12.304*
Sex (M)	-0.088	0.111	0.250
Treatment (DIS)	-0.232	0.134	4.795*
Treatment*Sex	0.115	0.180	0.478
Variances			
Natal family		0.015	
Residual		0.038	

* $p < 0.05$
